# Hydrophobic Surface Treatment for the Protection of Carparo Stone

**DOI:** 10.3390/polym18020237

**Published:** 2026-01-16

**Authors:** Marianna Potenza, Edoardo Verza, Federica Scigliuzzo, Sandro Meli, Antonella Casoli, Pier Paolo Lottici, Claudia Graiff, Laura Bergamonti

**Affiliations:** 1 Department of Chemistry, Life Science and Environmental Sustainability, University of Parma, Parco Area delle Scienze 17/A, 43124 Parma, Italy; marianna.potenza@unipr.it (M.P.); edoardo.verza@unipr.it (E.V.); federica.scigliuzzo@gmail.com (F.S.); sandro.meli@unipr.it (S.M.); antonella.casoli@unipr.it (A.C.); 2 Independent Researcher, 43124 Parma, Italy; pierpaolo.lottici@unipr.it

**Keywords:** Carparo stone, hydrophobic treatment, durability

## Abstract

The effectiveness of a hydrophobic coating based on TEOS/PDMS in protecting Carparo stone, a biocalcarenite characterized by high porosity and poor resistance to atmospheric agents and erosion, was evaluated. The hydrophobic treatment was applied over a pretreatment based on PMMA/ZrO_2_/SiO_2_ to promote a uniform distribution on the surface. Micro-tomography analyses demonstrate that pretreatment forms a homogeneous coating on the surface. Scanning electron microscopy investigation shows that the hydrophobic treatment based on TEOS/PDMS spreads across the entire surface. The coating is effective in reducing capillary water absorption, and the coated stones exhibit hydrophobicity, achieving contact angles > 140°. The coating has proven esthetically acceptable based on colorimetric tests. The durability of the treatments was evaluated through artificial aging consisting of rain cycles alternating with UV irradiation cycles. The contact angle tests carried out at the end of each cycle demonstrate that the protective coating is not leached and is still very effective. The new sustainable hydrophobic treatment can be successfully proposed for the protection of porous stones.

## 1. Introduction

The conservation of built heritage, especially monuments and historic buildings, due to their cultural and economic value, has attracted considerable attention in the scientific community for the search for new, effective and sustainable solutions [1,2,3,4,5,6].

Stone materials are generally subject to natural degradation, due to atmospheric agents and anthropogenic degradation, which can compromise the integrity and stability of the artworks [7,8,9].

Among stone materials, carbonate stones (marble, travertine and limestone) have been the most widely used [10,11] as structural and decorative elements in architectural applications since ancient times.

Water is known to be the main deterioration agent for building materials: the observed alteration phenomena, especially on porous limestones, are the transport of soluble salts, the dissolution of calcium carbonate and the development of microorganisms [12,13,14,15,16,17,18]. It is therefore necessary to implement preventive actions to mitigate damage due to water penetration [2,4,6,16,19,20,21]. Here we propose a new water-repellent product to be applied on a porous limestone, the Carparo stone.

Carparo is a calcarenite dating back to the Late Pleistocene, extracted in the coastal areas of Salento. It is predominantly a fine- to coarse-grained fossiliferous limestone, with varying degrees of compactness, porosity and toughness; it is sometimes associated with sandy-clayey deposits, primarily composed of quartz, feldspar and, to a lesser extent, pyroxene and iron aggregates, which are responsible for its yellowish color [22,23,24]. According to the Folk classification [25], it can be classified as a biocalcarenite.

Carparo has typically been used in Salento, Puglia (Italy), since ancient times as a building material thanks to its high workability [24,26,27]. Due to its high porosity, it is very susceptible to the appearance of efflorescence patinas and the growth of lichens, which cause an alteration of the color on the external facades of the monuments, turning them towards grayish tones [26,28,29]. The black crusts on the surface of the monuments made from Carparo are the main form of degradation [29].

To protect the monument’s surface, it is necessary to prevent water penetration and the deposition of pollutants, the main causes of stone deterioration.

Acrylic resins, silicone resins (alkylalkoxysilanes or polyalkyl alkoxysiloxanes), combinations of acrylic resins and silicones, and fluorinated polymers (fluorinated acrylates, perfluoropolyethers and perfluoropolyolefins) are the most used protective materials with water-repellent properties in the conservation of cultural heritage [16,30,31]. Each protective material has advantages and disadvantages: acrylic resins, for example, are very sensitive to thermal- and photo-oxidation [21]; alkoxysilanes show poor chemical compatibility with carbonate stones and a tendency to crack during drying [20,32]. Fluorinated polymers are the most effective to obtain superhydrophobic surfaces with high chemical stability and mechanical resistance [33,34]. However, the use of fluorinated compounds in the protection of exposed buildings is limited due to their poor adhesion to stone, their toxicity in the case of direct contact with the environment and their very high cost [35,36].

Hydrophobic treatments based on organically modified siloxanes (known as OrMoSils) are a new and effective alternative for use in the field of cultural heritage [37].

OrMoSils are a class of organic–inorganic hybrid materials in which the organic component can be chemically bonded to a silica matrix, giving flexibility to an inorganic network [38]. The presence of aliphatic groups also gives the material hydrophobic properties [39,40,41]. To increase the hydrophobicity and modify the wettability properties of surfaces, an effective strategy is to add nanoparticles [38,42], which can also improve their resistance to thermo- and photo-oxidation: nanoparticles such as SiO_2_, TiO_2_, ZrO_2_ and ZnO are typically added to hybrid systems [38,43,44,45].

The aim of this study was to obtain a highly hydrophobic coating and evaluate its ability to impart water-repellent properties to a highly porous stone. One of our objectives was to avoid the use of fluorinated polymers. Therefore, a hybrid hydrophobic coating based on OrMoSil with the addition of TiO_2_ nanoparticles was tested on Carparo samples. Before applying the hydrophobic coating, a pretreatment with PMMA added with inorganic ZrO_2_ and SiO_2_ particles was applied, which prevents yellowing of the methacrylate [46].

**Scheme 1:**
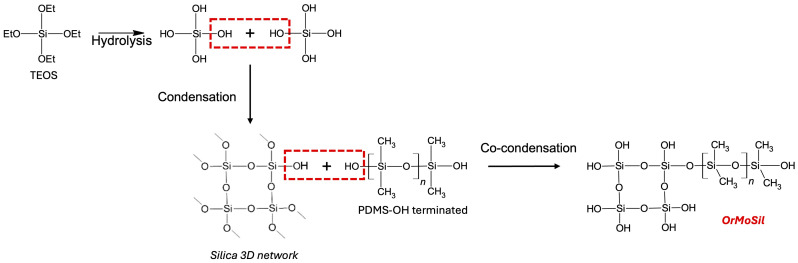
Synthesis of OrMoSil through sol–gel reaction between TEOS and PDMS-OH. The red box highlights the condensation of the silanol groups of TEOS followed by the co-condensation of the silanol groups of the alkoxysilane with the silanol groups of PDMS-OH.

OrMoSil was synthesized by the sol–gel process. The sol–gel process consists of the hydrolysis of an alkoxysilane, in this work TEOS, to form silanol groups, followed by the co-condensation of the silanol groups of the alkoxysilane with the silanol groups of the starting organic material, in our case PDMS-OH, as reported in Scheme 1 [47]. OrMoSil was mixed with TiO_2_ nanoparticles, obtaining a white-opalescent colloidal sol.

To evaluate the possibility of using this new treatment in the protection of cultural heritage, tests were carried out in accordance with the UNI-EN protection rules [48].

## 2. Materials and Methods

### 2.1. Synthesis of the Coatings

The coating formulation, based on PMMA/SiO_2_/ZrO_2_, applied on the stone surface as filler was prepared by a simple procedure, adding a sol based on SiO_2_/ZrO_2_, obtained by the sol–gel method, to a PMMA solution in a manner like that described in the literature [46]. PMMA pellets (average Mw~120,000, Sigma-Aldrich, St. Louis, MO, USA) were dissolved in a 50/50 mixture of ethylacetate (EtOAc)/tetrahydrofuran (THF) (Merck, Darmstadt, Germany) to obtain a 5% (*w*/*v*) polymer solution and stirred at 40 °C for approximately 4 h, until the solution became clear. In a separate flask, 0.025% (*w*/*v*) zirconyl chloride (ZrOCl_2_, Carlo Erba, Milan, Italy) was dissolved in ethanol and mixed with 0.025% (*w*/*v*) tetraethoxysilane (TEOS, Sigma-Aldrich, St. Louis, MO, USA). The sol was stirred at room temperature for 4 h to obtain a clear solution. The final product was obtained by slowly dripping the SiO_2_/ZrO_2_ suspension into the PMMA solution, and the mixture was stirred at room temperature for 4 h. The solution, hereafter referred to as **HyA**, was ready to be applied without further processing.

The hybrid organic–inorganic hydrophobic treatment was synthesized by the sol–gel method, following, with some modifications, the recipe reported in [31]. Briefly, in a round-bottom flask, TEOS (Sigma-Aldrich, St. Louis, MO, USA) as an inorganic precursor, oxalic acid dihydrate (OxA, Sigma-Aldrich, St. Louis, MO, USA) as a catalyst, AEROXIDE^®^ TiO_2_ P25 (Evonik Operations GmbH—Goldschmidtstraße 100, 45127 Essen, Germany), and ethanol (EtOH, Sigma-Aldrich) and distilled water as solvents were vigorously stirred for 140 min at room temperature to promote the hydrolysis. TEOS:EtOH:H_2_O:OxA:P25 was in the molar ratio 1:1.5:0.25:1 × 10^−5^:0.5 × 10^−4^. Hydroxyl-terminated polydimethylsiloxane (PDMS-OH, Sigma-Aldrich, St. Louis, MO, USA) in a ratio of TEOS:PDMS = 1:1 (*v*/*v*) was slowly added to the previous solution and the suspension was stirred overnight to promote the condensation between the silanol groups of TEOS and the hydroxyl groups of PDMS. The formulation, named **HyB**, was transparent and ready to apply.

**Scheme 2:**
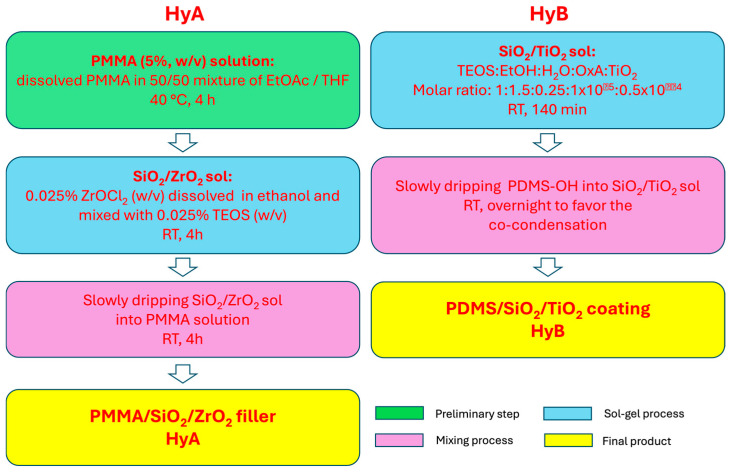
Preparation of the **HyA** filler and of the **HyB** hydrophobic coating.

The flowchart for **HyA** and **HyB** preparation is reported in Scheme 2.

For comparison purposes, a commercial hydrophobic product (High Materials Innovation, (HMI), s.r.l., Parma, Italy), a trifunctional organic compound based on a water-based fluoroalkyl functional oligosiloxane, hereinafter referred to as **HyC**, was tested.

### 2.2. Carparo Sample Preparation

According to the UNI 10921:2001 standard [48], twenty-one Carparo samples measuring 5 × 5 × 1 cm^3^, obtained from a single block, were brought to constant mass in an oven at (60 ± 2) °C. Sixteen samples (ten for the tests without aging and six for the aging tests) were pretreated with the **HyA** filler, oven-dried at 60 °C for about 16 h and then left to cool in a desiccator before further treatment. Eight pretreated samples were brush-coated with **HyB** and eight with **HyC**. All samples were left to dry in an oven at 60 °C overnight. For comparison, five untreated Carparo samples were left as reference for the tests before aging. Samples for petrographic, morphological and elemental analyses, Raman and FTIR spectroscopies and micro-CT were appropriately extracted from the same block and cut to the required dimensions. The number of samples, their size, the analyses/tests performed, the number of measurements and the standard rules are summarized in Table 1.

### 2.3. Characterizations

Carparo stone was petrographically analyzed by using a Nikon Eclipse E400 POL (Nikon Corporation, Minato-Ku, Tokyo, Japan) polarized optical microscope to characterize its mineralogical and microstructural features (grain size, matrix, cement and porosity). The microscopic features were carried out under transmitted light on thin sections (~30 μm). An overview of the bulk microstructure of the rock was performed by a Nikon SuperCoolScan 5000 ED scanner (Nikon Corporation, Minato-Ku, Tokyo, Japan), with a scanning resolution on the whole thin section of 4000 × 6000 pixels.

Morphological observations before and after the treatments and elemental analysis to study the distribution of the coatings on the raw stone surface were performed by means of a SEM-EDS system: a Jeol 6400 scanning electron microscope (Jeol Ltd., Tokyo, Japan) equipped with an Analytical Link Si(Li) Energy Dispersive System Detector, with INCA V7.2 software (Oxford Instruments, Abingdon-on-Thames, UK).

Raman measurements were carried out in a backscattered geometry with a LabRAM micro-spectrometer (Jobin Yvon Horiba, Kyoto, Japan) equipped with an Olympus BX40 microscope (Olympus Corporation, Tokyo, Japan). The 632.8 nm line of a 15 mW He–Ne laser was used, and the spectra were collected, at room temperature, by a long-working-distance ×50 microscope objective lens.

FT-IR spectra were recorded in ATR mode in the range 4000–400 cm^−1^, with a spectral resolution of 4 cm^−1^, by means of a Thermo-Nicolet Nexus spectrometer (Thermo Fisher Scientific, Madison, WI, USA) equipped with a Thermo Smart Orbit ATR diamond accessory. 

Thermogravimetric analysis was performed to evaluate the thermal stability of the synthesized hybrid coatings (**HyA** and **HyB**) using a Perkin Elmer TGA8000 instrument (PerkinElmer, Waltham, MA, USA). Measurements were carried out on about 10–15 mg of sample in air, at a heating rate of 10 °C/min and a flow rate of 20 mL/min, in the temperature range 30–900 °C. The derivative of the weight loss curve (DTG) was recorded.

To investigate the morphological changes in the internal structure, a Carparo sample was scanned using a micro-CT scanner (NeoScan N60, Mechelen, Belgium) with the following parameters: voltage 63 kV and current 761 µA, with a 0.50 mm copper–aluminum filter and an image pixel size of 7.3 µm. Scanning options were performed with a rotation step of 0.2°, an average of 8 frames and a rotation angle of 360°. During the reconstruction procedure, a misalignment of 4 pixels, a smoothing of 4 pixels and a beam hardening of 60% were applied, with a gray scale of 0.108–1.032. Local size analyses were also performed using the same software, during the processing procedure, applying an image to a binary scale of 80…225, before the Shrink Wrap operation, with a 2D probe radius of 5 pixels. The reloaded images were subsequently binarized with a 0…80 scale before the analysis. All parameters were calculated in 3D and compared before and after treatment on the same sample to study the changes due to the application of the **HyA** filler. Given the transparency of the **HyA** treatment under tomographic analysis, a barium chloride solution (1%) was added as a radiopaque tracer for X-ray imaging.

The chromatic changes in the stone surface appearance due to the **HyB** and **HyC** treatments were evaluated according to UNI EN 15886:2010 rules [49] by colorimetric analysis performed by a SpectroDens Premium colorimeter, software 8.08/01.06.10, version hardware 2, serial number B110040 (Techkon GmbH, Königstein, Germany). The results were averaged from five samples (six points per sample) for each case, before and after treatments. The color difference ΔE* due to the coating was measured as

(1) $${∆E}^{*}=\sqrt{{∆L}^{*2}+{∆a}^{*2}+{∆b}^{*2}\;}$$

where ΔL* is the change in lightness and Δa* and Δb* are the changes in the colorimetric coordinates a* (red/green) and b* (yellow/blue) in the CIELAB space.

To evaluate the hydrophobic properties of the coatings, static contact angle (CA) measurements were performed by the Dataphysics OCA (Optical Contact Angle) 20 instrument (NG Labtec Srl, Bergamo, Italy), using the sessile drop method, with 5 µL drops applied via a needle to the stone surface before and after treatment, according to UNI EN 15802:2010 [50].

The water absorption by capillarity was estimated on five untreated and five **HyB**- and **HyC**-treated samples, according to the UNI EN 15801:2010 standard [51]. The dried samples were placed on a Whatman multilayer paper (1 cm thick) soaked with deionized water. Samples were weighed at *t_i_* = 10 min, 20 min, 30 min, 60 min, 2 h and 6 h and at 24 h intervals until the difference between two successive weighings was <1% of the mass of water absorbed by the sample. The amount of absorbed water per unit area *Q_i_* (kg/m^2^) is determined by *Q_i_* = [(*m_i_* − *m*_0_)/*A*], where *m_i_* and *m*_0_ are the mass of the sample at time *i* and the initial time, respectively, and *A* is the area of the sample. The calculated *Q_i_* values were reported in a graph as a function of the square root of time (t_i_^1/2^), as, in accordance with a well-known capillarity absorption model [53], one expects a linear relationship between the absorbed water and the square root of time in short time periods. The value of the capillary water absorption coefficient *AC* (kg/(m^2^s^1/2^)) was obtained from the slope of the linear fit (using at least 5 successive aligned points) of the capillarity curve (*Q_i_*).

The water vapor permeability test was performed on five untreated stone samples and on five samples for each treatment, following the UNI EN 15803:2010 standard [52]: the 5 × 5 × 1 cm^3^ samples, preliminarily conditioned at 23 ± 2 °C and 50 ± 5% relative humidity (RH) for 24 h, were sealed with paraffin along the edges and fixed onto PVC containers, partially filled with a known amount of water. Then, the sealed containers were placed in a climate chamber (RH 50 ± 5% and T = 23 ± 2 °C) and weighed every 24 h until constant vapor flow through the stone was reached (i.e., the condition ((Δ*M*_i_ − Δ*M*_i–1_)/Δ*M*_i_) × 100 ≤ 5, where Δ*m_i_* is the difference between two successive weighings). The water vapor transmission rate *WVTR* = *M_v_*/*At* (g/(m^2^d)) is defined as the mass *M_v_* (g) of water vapor passing through the surface *A* (m^2^) in the time *t* (24 h) as reported in [20]. The reduction percentage vapor permeability (*RVP*%) induced by the treatments was evaluated as *RVP*% = ((*M_uv_* − *M_tv_*)/*M_uv_*) × 100, where *M_uv_* and *M_tv_* are the mass of water vapor through the untreated and treated stone, respectively, measured at the steady state, as proposed by Raneri [5]. The steady state was reached in 120 h for both the treated and untreated samples.

### 2.4. Artificial Aging

The artificial aging test was performed only on treated Carparo stone (three samples for each case). Four cycles of exposure to rain and artificial solar radiation were performed. Each 24 h cycle consisted of 620 mm of water (i.e., the average annual rainfall in Salento) dropped on the samples, for 6 h, in the form of water mist, followed by 16 h of artificial solar irradiation under an Osram Ultra Vitalux lamp (300 W) (in UVA + B ∼ 5.5 mW/cm^2^ and in the 300–800 nm range ∼ 56 mW/cm^2^ [46]) and 2 h of darkness in room conditions. The test was carried out in a closed container (45 × 35 × 30 cm^3^) equipped with a 45° inclined plane for housing the samples and with a lateral nebulization system with two nozzles. The photo-oxidation stability of the coatings was assessed by means of colorimetric measurements recorded before and after aging. The hydrophobic properties of the coatings were tested by measuring the static contact angle at the end of each cycle. Table 2 summarizes the number of samples, number of measurements, and tests performed to verify the coating’s stability under the artificial aging.

## 3. Results

### 3.1. Coating Characterization

#### 3.1.1. Fourier Transform Infrared (FTIR) Spectroscopy

**Figure 1:**
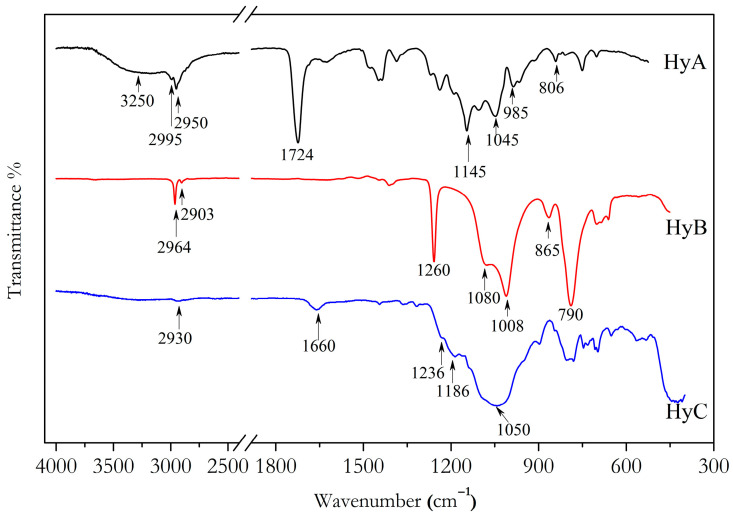
FTIR spectra of **HyA** and of the hydrophobic coatings **HyB** and **HyC**.

The **HyA** filler and the **HyB** and **HyC** hydrophobic coatings were characterized by FTIR spectroscopy. The spectra are reported in Figure 1.

In the FTIR spectrum of **HyA** (PMMA/SiO_2_/ZrO_2_ composite film), the typical PMMA stretching vibrations of methyl groups at (2995 cm^−1^ and 2950 cm^−1^) and the stretching vibrations of the ester groups (C=O at 1724 cm^−1^ and C-O at 1145 cm^−1^) are observed [37]. The slight shift to low wavenumbers of the carbonyl absorption band indicates the possible interaction between the inorganic components, in particular ZrO_2_, and the PMMA matrix, due to the acceptance of electrons by the Zr atoms, which results in a strong interaction between the ZrO_2_ and the polymer [54]. The inorganic components introduce the broad and strong absorption band centered at about 3250 cm^−1^, due to the O-H stretching vibrations of SiO-H and ZrO-H, from the incomplete condensation in the sol–gel process. The Si-O stretching vibration, usually located at about 1070 cm^−1^, is shifted toward lower wavenumbers at 1045 cm^−1^, suggesting the formation of Si-O-Zr bonds. The stretching vibration of Si-OH groups is found at 985 cm^−1^ and the O-Zr-O bending vibration at 806 cm^−1^ [55].

In the spectrum of the **HyB** treatment, complete cross-linking between TEOS and PDMS and a complete condensation of TEOS is demonstrated by the absence of bands in the regions of O-H stretching and bending [54]. The characteristic bands of the PDMS chains are at 2960 cm^−1^ and 2903 cm^−1^, assigned to the stretching vibrations of the methyl group, and at 1260 cm^−1^ and at 790 cm^−1^, both attributed to the Si-CH_3_ bond [54,55,56]. The strong bands assigned to the asymmetric and symmetric Si–O–Si stretching vibrations are found at 1080 cm^−1^ and 1008 cm^−1^, respectively. The band at 865 cm^−1^ results from the condensation reaction of TEOS, following the progress of the sol–gel process, and from the reaction between TEOS-PDMS, which leads to the formation of the hybrid organic–inorganic 3D network [57,58].

**HyC**, based on fluoroalkyl functional oligosiloxane, shows the characteristic vibrations of the siloxane network at around 1050 cm^−1^, features due to the fluoroalkyl groups at 1241 and at 1186 cm^−1^, indicative of the C-F bond, and the vibration of -NH_2_ at 1660 cm^−1^ [59,60].

#### 3.1.2. Thermal Analysis (TGA/DTG)

**Figure 2:**
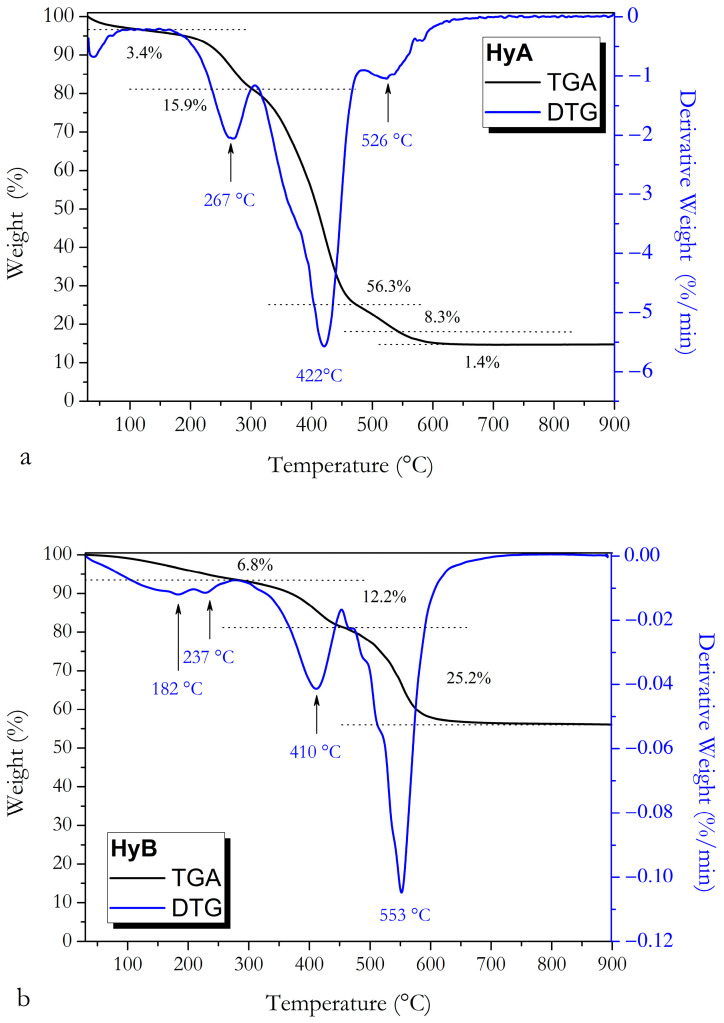
TGA and DTG curves of **HyA** based on PMMA/ZrO_2_/SiO_2_ (**a**) and of **HyB** based on PDMS/SiO_2_/TiO_2_ (**b**).

Figure 2 shows the thermograms (TGA) of weight loss as a function of temperature (30–900 °C) together with the corresponding differential thermogravimetric (DTG) profiles of the synthesized **HyA** and **HyB** coatings.

As reported by several authors [46,54,55,61,62], the effective dispersion of inorganic nanoparticles in the polymer matrix increases the thermal stability of nanocomposites by limiting the mobility of the polymer chains. Thermal stability of the PMMA/ZrO_2_/SiO_2_ composite (**HyA**, Figure 2a) is improved with respect to the pure PMMA by the addition of the inorganic component. Pure PMMA exhibits two degradation steps as found by Wang et al. [55]: a small weight loss at 160 °C due to the cleavage of the head-to-head bonds, and a single weight loss of about 95% between 300 and 400 °C, with a peak at 382 °C as determined by DTG, corresponding to the cleavage of the unsaturated vinyl ends and the random cleavage of the polymer backbones. The hybrid coating **HyA** shows four degradation steps: The first one begins with head-to-head bond cleavage between 100 °C and 200 °C (not identifiable in the DTG curve). The contribution of head-to-head bonds to mass loss is relatively small and appears as a shoulder of the second peak, at 267 °C, caused by the scission of the end groups. The third step, due to the degradation of the PMMA matrix, is significantly improved: it occurs at 422 °C. This increase likely results from the formation of an interpenetrating network and cross-links between the organic and inorganic components, which can inhibit the movement of free radicals and their attack on the PMMA backbone, causing its scission [55]. The last step, evidenced by the small peak at 526 °C, could be due to the degradation of the SiO_2_ network [55,56,61,62].

As reported by Camino et al. [63,64], thermo-oxidative degradation of PDMS occurs in two steps: at 339 °C and at 400 °C. In both steps, the main degradation products are the result of the decomposition of the organic groups of PDMS. As for the **HyA** coating, the addition of the inorganic component also increases the thermal stability of the **HyB** coating. The thermogravimetric profile of the **HyB** composite (Figure 2b) shows three steps associated with weight loss. The first loss (about 7%) occurs in the range 100–300 °C and corresponds to two events: the first at 182 °C may arise from residual solvent trapped in the pore structure; the other, at 237 °C, is caused by the advanced condensation process of the Si-OH groups of TEOS and PDMS to form Si-O-Si, with elimination of volatile byproducts [65,66]. In the TGA curve, two mass reductions are observed in the range 350–700 °C: one at 410 °C (12%) and one at 535 °C (25%). The weight loss can be attributed to the thermal decomposition of PDMS (at a higher temperature than that of the pure PDMS network), which forms cyclic oligomers due to the cleavage of the Si–O bonds during heating, which are completely decomposed above 500 °C [63,64]. These results are indicative of the enhancement of the thermal stability of the hybrid composite.

### 3.2. Characterization of the Stone Samples

#### 3.2.1. Transmitted-Light Polarized Optical Microscopy

**Figure 3:**
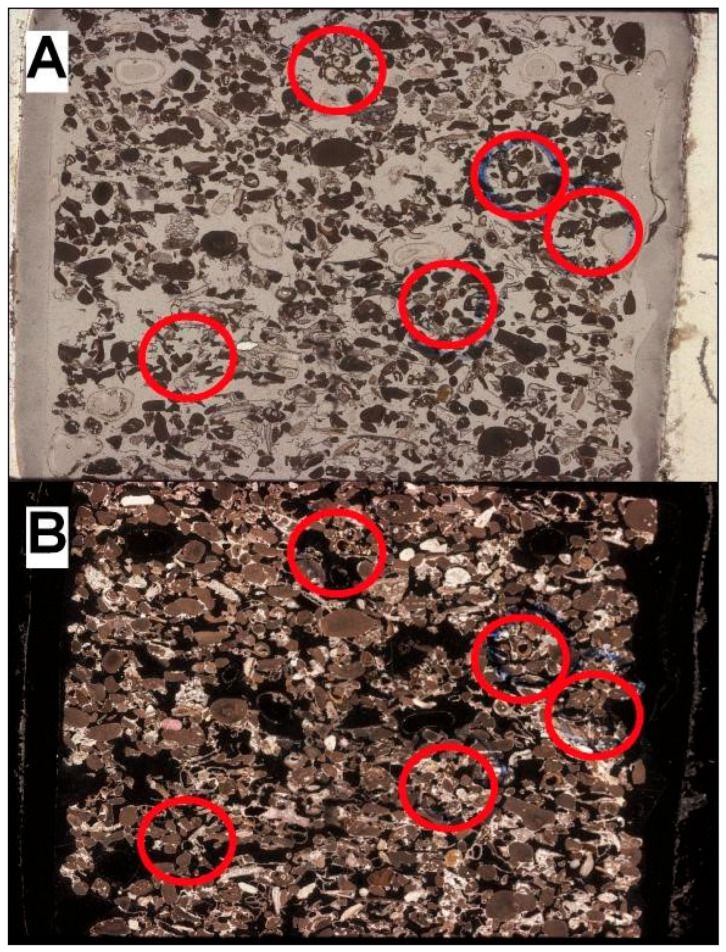
Overall scanning of the thin section of a representative sample of Carparo stone. (**A**) Plane polarized light, PPL; (**B**) crossed polarized light, XPL. Red circles highlight sites in which SEM analyses were performed. The dominating gray pinkish color (“interference color”) in the XPL image underlines the high predominance of a calcitic component in Carparo stone. Black sectors in (**B**) allow the porosity degree of the stone to be estimated. Field of view: 58 × 28 mm.

Overall scanning by polarized optical microscopy (OM) of a whole thin section displays a highly porous structure (Figure 3), with a dominant calcareous component, represented by fossil fragments and micrite clasts, among which minor siliceous phases are present (mainly quartz, with minor feldspars, plagioclase and pyroxenes). Dispersed, oxidized ferric aggregates in the stone confer their yellowish, dominant color. Carparo stone can be classified as a calcarenite. The microstructure of the rock appears heterogeneous, with portions formed by fossil fragments, micrite aggregates and quartz crystals; it has a dominantly coarse grain size (100 mm to 2–3 mm).

**Figure 4:**
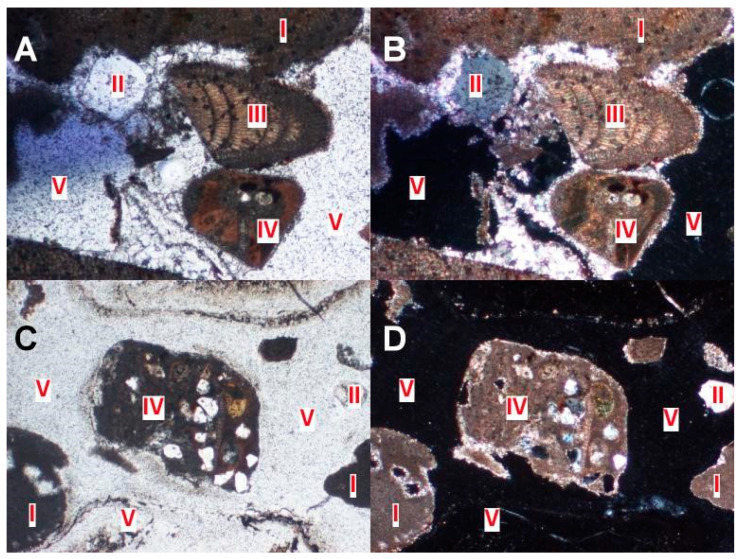
Microstructure details of Carparo limestone: (**A**,**C**) PPL, (**B**,**D**) XPL. I: micrite clasts; II: quartz clasts; III: fossil fragments, unclassifiable through a single thin section view; IV: quartz-bearing (white fields inside) micritic siltite fragments; V: pores. Field of view: 10 × 7.5 mm).

Another example, even more illustrative of the heterogeneous microstructure of the rock, is shown in Figure 4, where one observes clasts formed by micrite, millimetric fossiliferous fragments and small quartz crystals.

#### 3.2.2. Raman and FTIR Spectroscopies

**Figure 5:**
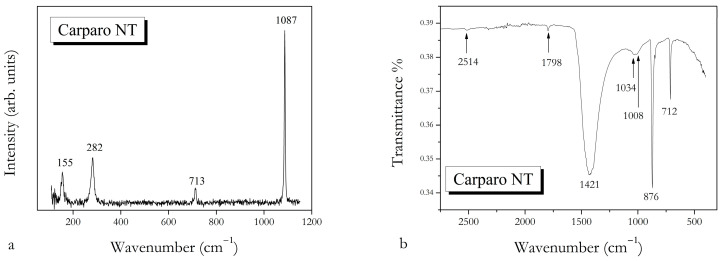
(**a**) Raman and (**b**) FTIR spectra of untreated Carparo stone.

The Carparo stone samples were characterized by FTIR and Raman spectroscopies (Figure 5). As is well known, carbonate minerals are characterized by the vibrations of (CO_3_)^2−^ groups that have four normal modes: the Raman-active symmetric stretching (υ_1_) modes (in the 1000–1100 cm^−1^ range), the IR-active out-of-plane bending (υ_2_) modes (870–890 cm^−1^), the asymmetric stretching (υ_3_) modes (1400–1450 cm^−1^) and the in-plane bending (υ_4_) modes (700–720 cm^−1^); the υ_3_ and υ_4_ modes are both Raman- and IR-active [67,68].

In the Raman spectrum (Figure 5a), recorded up to 1200 cm^−1^, the strong peak at 1087 cm^−1^ and the one at 713 cm^−1^ are attributed to the υ_1_ and υ_4_ modes of the (CO_3_)^2−^ groups, respectively. The peaks at 152 and 280 cm^−1^ arise from translational and rotational oscillations of the (CO_3_)^2−^ groups. The peaks found are characteristic of calcium carbonate (CaCO_3_) in calcite phase, confirming that calcite is the main mineralogical component of the stone. In the FT-IR spectrum (Figure 5b), typical contributions from calcite (CaCO_3_) centered at 1421, 875 and 712 cm^−1^, which correspond, respectively, to the υ_3_, υ_2_ and υ_4_ modes, are recognizable. Besides the first-order modes, the υ_1_ + υ_4_ (1798 cm^−1^) and 2 υ_2_ + υ_4_ (2514 cm^−1^) combination modes are observed [67,68]. The bands centered at 1034 cm^−1^ and 1008 cm^−1^ are due to silicate phases (Si–O vibrations), probably resulting from the clay mineral content, in agreement with the petrographic data.

### 3.3. Characterization of Coated Stones and Effects of the Coatings

#### 3.3.1. FTIR Spectroscopy

**Figure 6:**
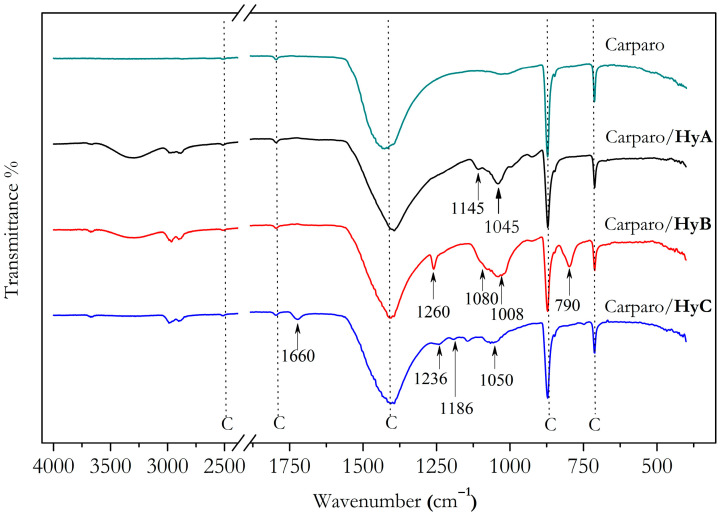
FTIR spectra recorded on untreated, pretreated (**HyA**) and treated (**HyB** and **HyC**) Carparo stones. C indicates the vibrational peaks of calcite.

FTIR spectra of treated Carparo stone are reported in Figure 6, compared with untreated stone. All FT-IR spectra are dominated by typical contributions from calcite (CaCO_3_). The FTIR spectra recorded on the surface of the coated stones clearly show the features of the different treatments applied, as discussed in the Section 3.1.1.

#### 3.3.2. Scanning Electron Microscopy (SEM) and Energy Dispersive Spectroscopy (EDS)

The morphology of the treated and untreated stones was studied using scanning electron microscopy (SEM), performed to observe the arrangement of the coatings on the surface and verify the chemical composition.

**Figure 7:**
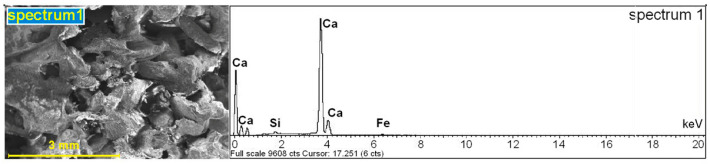
SE image and average EDS spectrum on the whole area of the untreated Carparo sample.

Figure 7 shows the images produced by the secondary electrons and the corresponding EDS spectra, averaged over the entire surface of interest of the untreated sample.

Carparo has a highly porous microstructure, with grains of various sizes. EDS elemental analysis shows calcium as the rock’s main constituent. Calcium correlates with the calcareous component consisting of the mineral calcite (identified by vibrational Raman spectroscopy and FTIR), fossil fragments and micrite clasts. Elemental analysis also reveals small amounts of silicon (Si) as a minor component, likely related to the clay minerals, quartz and feldspar (identified by petrographic analysis). Traces of Fe are present, probably due to small iron aggregates in the stone which give it its characteristic yellowish color.

**Figure 8:**
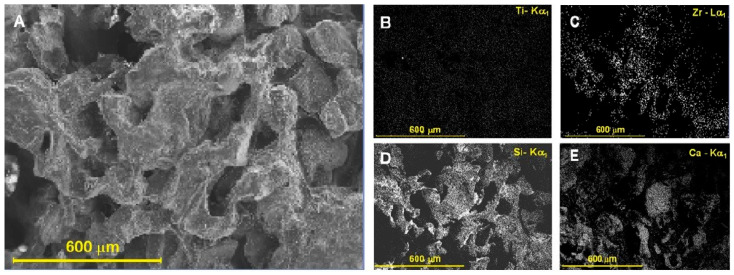
Qualitative SEM-EDS grayscale maps of the surface distribution of Ti, Zr and Si in the Carparo sample treated with **HyB**. (**A**) Morphological SE image. (**B**–**E**) Elemental maps; the brighter the color, the higher the concentration.

**Figure 9:**
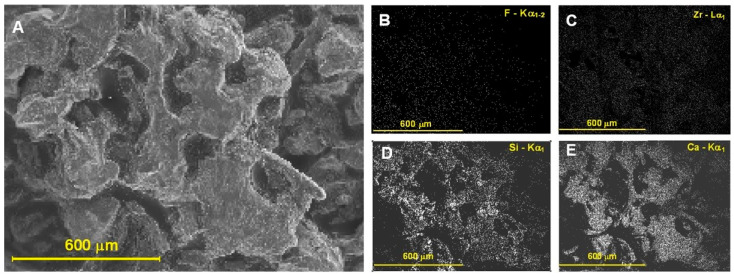
Qualitative SEM-EDS grayscale maps of the surface distribution of Si, Zr and F in the Carparo sample treated with **HyC**. (**A**) Morphological SE image. (**B**–**E**) Elemental maps; the brighter the color, the higher the concentration.

SEM images were recorded on the surface of the **HyB** (Figure 8)- and **HyC** (Figure 9)-coated stones to estimate the homogeneity of the hybrid coating. The elemental distribution of the coatings on the surface of the samples was estimated by collecting the primary X-rays emitted by the sample from an area 1.2 × 0.8 mm wide.

Compared to the untreated stone, the coating exhibits a homogeneous distribution, attenuating the roughness of the substrate, which appears smoother. From the elemental maps of Si, Zr and Ti present in **HyB** and Si, Zr and F present in **HyC**, the treatment mirrors the microstructure of the stone, distributing itself uniformly.

#### 3.3.3. Microcomputed Tomography (Micro-CT)

**Figure 10:**
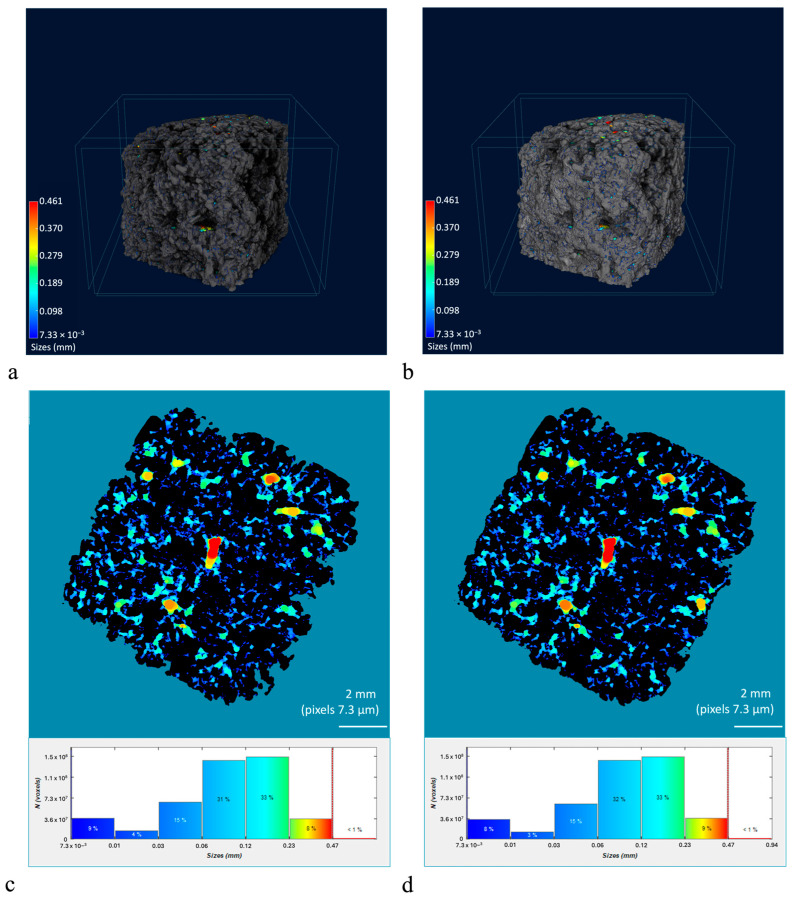
Three-dimensional reconstruction of the untreated (**a**) and **HyA**-treated (**b**) Carparo, with a colorimetric scale representing porosity derived from local size analysis. Local size analysis of the same slice from the untreated (**c**) and treated (**d**) samples and their histograms.

The Micro-CT technique relies on a series of X-ray radiographs taken at different rotation angles, from which cross-sectional images of the sample can be reconstructed [69]. This nondestructive technique has already been demonstrated to be an important tool for stone conservation [70], allowing the investigation of the internal structure of materials. From the 3D reconstructions shown in Figure 10, it can be observed that compared to the untreated sample, which presents a high roughness (Figure 10a), the stone after the application of the **HyA** filler (Figure 10b) shows a smoother and more uniform surface morphology. This suggests that the treatment mainly affects the outer layers, resulting in the formation of a thin surface coating. The 3D reconstruction of the untreated Carparo, combined with the pore mapping obtained through the local size analysis of the entire volume, highlights that the treatment did not penetrate inside the stone, and the void distribution mirrors that of the treated sample (Figure 10c,d). Tomographic analyses of the Carparo samples before and after treatment clearly show that the PMMA does not penetrate the internal porous network of the rock and does not modify its intrinsic porosity; consequently, the histograms in Figure 10c,d for both samples show no significant variations.

**Figure 11:**
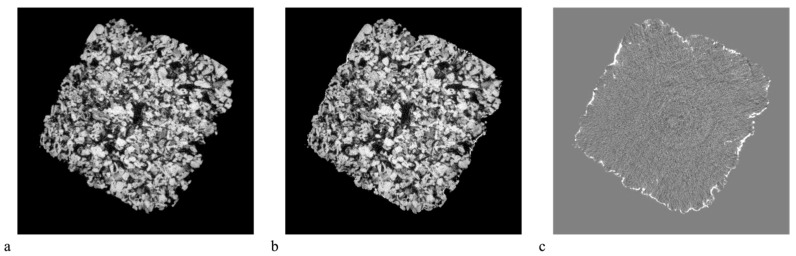
Comparison of the untreated (**a**) and **HyA**-treated (**b**) Carparo slices used for the overlay in the Neofusion software. (**c**) Superimposition of the same slices (**a**,**b**) performed using the NeoFusion software, version 1.0.0.

Using the NeoFusion program, the sections comprising the untreated (Figure 11a) and treated (Figure 11b) samples were superimposed (Figure 11c) to quantitatively evaluate the deviations. As illustrated in Figure 11c, the central portion appears almost uniform in terms of grayness, indicating a perfect congruence between treated and untreated Carparo in terms of internal porosity and morphology. On the contrary, marked discrepancies are visible along the edges, where a continuous and well-distributed contour covering the entire surface is clearly visible as a direct consequence of the treatment.

#### 3.3.4. Measurement of Color Surface 

A fundamental requirement for treatments applied to materials of historical and artistic interest is that they do not alter the color of the surfaces. To test the harmlessness of the hydrophobic coatings with respect to the esthetic appearance of the stone, colorimetric measurements were performed, according to UNI EN 15886:2010 [49]. As observed by petrographic analyses, Carparo is characterized by a heterogeneous texture. The heterogeneity of the stone causes color tones to vary from one point to another within the same sample, from a light-yellow color to a more intense yellow. This makes colorimetric measurements extremely difficult and may result in an overall colorimetric variation slightly greater than the actual one. Nonetheless, analyses performed before and after the treatments show acceptable color variations, although close to the ΔE* = 5 limit established by the regulations [15]. Both treatments show an average colorimetric variation of ΔE* ≈ 4 (Table 3). Analyzing the differences in detail, it can be noted that in the case of the **HyB** treatment, there is a small increase in the b* coordinate (which goes from 18 ± 1 to 22 ± 1), which causes a slight yellowing, while in the case of the **HyC** treatment, the L* coordinate varies, going from 68 ± 3 to 72 ± 1, causing a lightening of the stone. The a* coordinate remains nearly constant for both treatments.

#### 3.3.5. Determination of the Static Contact Angle 

Static contact angle (CA) measurements were performed according to UNI EN 15802:2010 [50].

**Figure 12:**
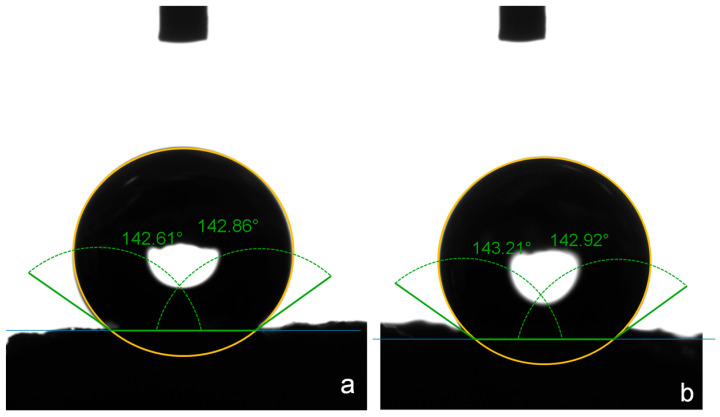
Images of representative static contact angles acquired on **HyB** (**a**) and **HyC** (**b**).

These measurements allow for a rapid assessment of the local hydrophobicity of the surface. Due to the high porosity, it was not possible to measure contact angles on the untreated stone. The **HyA** primer, as observed by micro-CT analysis, forms a homogeneous film on the surface that occludes the stone’s asperities. The contact angle measured on the stone with **HyA** alone was 93 ± 4°. The **HyB** coating produces an improvement in the water repellency of the sample surfaces. The contact angle is comparable to that of the commercial **HyC** coating. Figure 12 shows the droplet images recorded on samples coated with **HyB** and **HyC**: the contact angle values are greater than 140° for both coatings (Table 3).

#### 3.3.6. Water Absorption by Capillarity

**Figure 13:**
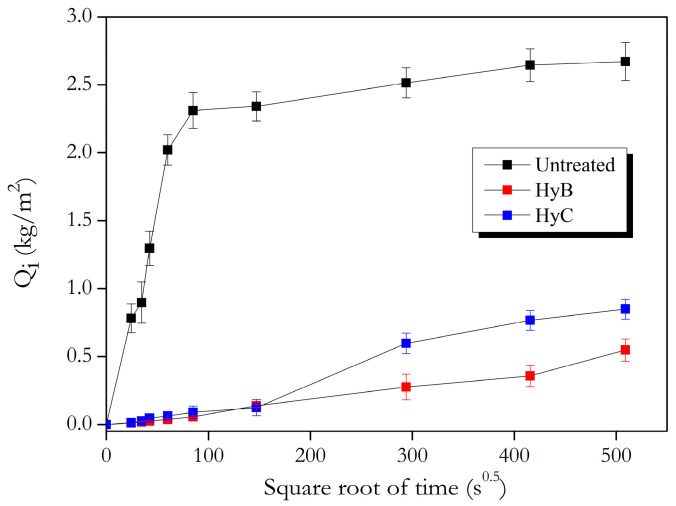
Absorbed water *Q_i_* reported as a function of the square root of time.

The presence of water is one of the main factors in the deterioration of stone materials. Capillary rise is the most common mechanism for water penetration into building materials. The effectiveness of the hydrophobic coating in protecting the Carparo stone was tested by measuring the water capillary absorption before and after treatment according to UNI EN 15801:2010 [51]. Figure 13 shows the amount of water absorbed by the samples per unit area *Q_i_* (kg/m^2^) at time *t_i_* plotted as a function of the square root of time (in seconds). As can be seen from the graphs, at the end of the test (72 h), the treatment induced a significant reduction in water absorption of about 80%. As expected, the untreated Carparo specimens, due to their high porosity, absorbed about 85% of the amount of water absorbed during the entire test within the first two hours. The treated specimens, at the same time, have a negligible absorption. This improvement is due to the ability of hydrophobic treatments to form a barrier to water penetration. The treatment influences the capillary absorption kinetics compared to untreated stones and reduces water absorption, as evident from the capillary absorption coefficient *AC* which represents the rapidity of capillary rise in short time periods. The coefficient *AC* was obtained as the slope of the line fitted to the *Q_i_* curve versus t^1/2^ in the range 0–120 min. The *AC* (in kg/(m^2^ s ^½^)) was 6.3 × 10^−4^ ± 2 × 10^−5^ and 1.0 × 10^−3^ ± 5 × 10^−4^ for **HyB** and **HyC**, respectively, and 0.029 ± 0.001 for the untreated Carparo stone. Results are summarized in Table 3.

#### 3.3.7. Water Vapor Permeability

**Figure 14:**
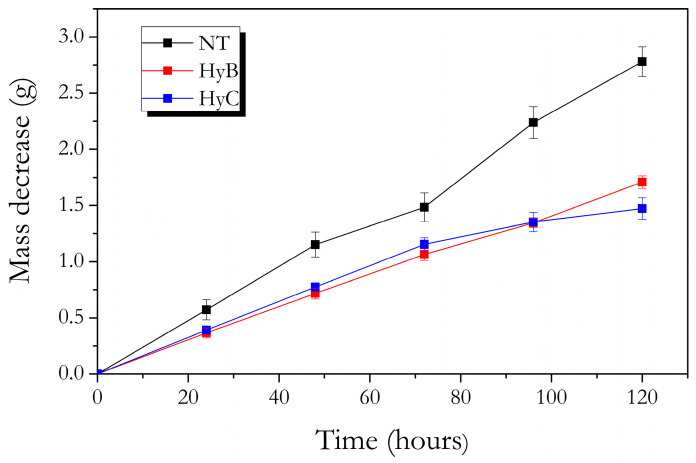
Mass decrease (i.e., variation in the cumulative vapor mass transmitted by the untreated and treated stones), measured during the water vapor permeability test.

Carparo stone is characterized by high breathability due to its porous structure. A comparison between the transmission of water vapor of untreated and treated specimens is plotted in Figure 14.

The curves in Figure 14 show that the coated stones experience a significant reduction in vapor flow, especially in the stone treated with the fluorinated hydrophobic filler **HyC**. It has been reported by other authors that fluorinated hydrophobics, due to their high water repellency, can cause a drastic reduction in permeability [30,71]. The reduction percentage vapor permeability *RVP*% for **HyB** is 40 ± 2%, while for **HyC** the reduction is 48 ± 1%. Even a small variation in RVP%, as in HyB, could allow for greater breathability of the stone and be useful to prevent moisture entrapment.

The water vapor transmission rate (*WVTR*) of the coated stones is 76 ± 3 g/(m^2^d) and 68 ± 2 g/(m^2^d) for **HyB** and **HyC**, respectively, significantly lower than that recorded for the untreated stone, 130 ± 2 g/m^2^d. According to [72], the water vapor transmission rate can be grouped into three classes:

Class I: high water vapor transmission rate if V > 150 g/(m^2^d);

Class II: medium water vapor transmittance if 15 < V < 150 g/(m^2^d);

Class III: low water vapor transmittance if V < 15 g/(m^2^d).

**HyB** and **HyC** have values in the Class II range, the same as the untreated stone.

Results are summarized in Table 3.

The reduction in water permeability in treated stones compared to untreated ones could be attributed to the modification of the surface porous microstructure, mainly due to pretreatment with the hybrid product **HyA**. The hybrid filler, as observed by μCT investigations, coats the surface homogeneously, causing the stone’s roughness to smooth out. This results in a marked reduction in capillary water absorption, as noted in the previous paragraph, but obviously also leads to a limitation of the passage of water vapor within the stone, which results in a reduction in breathability. It is not uncommon for hydrophobic treatments to result in a severe reduction in breathability when used on porous substrates [5,40].

Although the reduction in VPR is higher than the recommended one (<25%) [73], the stones maintain good breathability, which may represent a good compromise compared to the high protection offered by the covering.

### 3.4. Artificial Aging

The artificial aging test was performed as explained in the Section 2.4 on three **HyB**- and **HyC**-treated samples. To evaluate the weathering stability of the hydrophobic treatments at the end of the four cycles of rain, irradiation and darkness, contact angle and colorimetric parameters were recorded on the stones and the results were compared to those obtained on coated samples before the aging cycles (Table 4). It was decided to check only static contact angle measurements because they provide a rapid and effective assessment of the local hydrophobicity of the surface, rather than replicating the barrier properties against bulk water absorption. In addition, colorimetric measurements are essential for establishing the coating resistance to photo-oxidation responsible for color alterations of the artifacts. 

**Figure 15:**
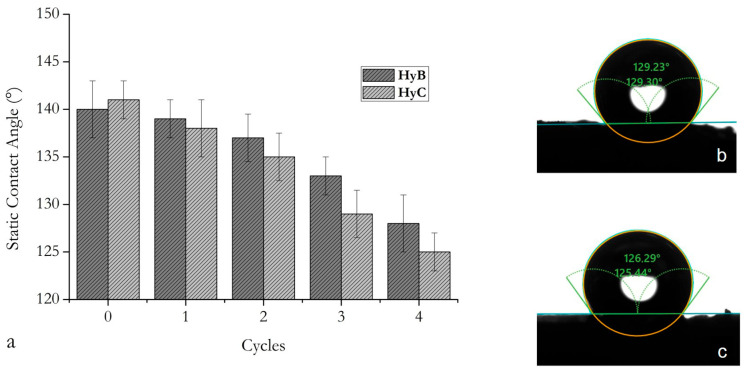
Contact angle**s** for each aging cycle (**a**); representative measurements of static contact angle on **HyB** (**b**) and **HyC** (**c**) at the end of the artificial aging test.

Figure 15 shows the contact angle variation during aging cycles (a) and water droplets deposited on the surface of the treated samples **HyB** (b) and **HyC** (c) at the end of the aging cycles.

As can be seen in the figures, the sample treated with **HyB** gives a contact angle ~129°, while for the sample treated with **HyC**, the contact angle is ~126°. Despite the stress experienced during the aging test, the treatments maintain their water repellent efficacy. The inorganic component of the **HyB** forms a 3D network that interacts with the siloxane component of the **HyA** pretreatment, allowing the coating to remain well adhered to the surface even after repeated rain cycles.

To assess the stability of the coatings, colorimetric coordinates were measured on the coated samples before and after the aging. Even from a colorimetric point of view, despite having undergone photooxidative stress, the samples did not show significant color changes. The **HyB** coating undergoes a slight yellowing due to a shift towards more positive values of the colorimetric coordinate b* (from 22 ± 1 to 24 ± 1). However, the overall ΔE* is not perceptible to the eye (ΔE* less than 3). The **HyC** coating remains essentially unchanged (L* = 73 ±1, b* = 16 ± 1).

## 4. Conclusions

The objective of this work was to test protective surface treatments for the conservation of Carparo, a biocalcarenite widely used in the Baroque period in the construction and architecture of the cities of Salento. This stone is highly sensitive to the effects of water, being highly porous. To improve the stone’s durability, a non-fluorinated hydrophobic coating based on organically modified siloxane was proposed.

To allow the hydrophobic coating to homogeneously coat the surface, a PMMA/ZrO_2_/SiO_2_ pretreatment was applied to the samples. A comparison was made between the new hydrophobic coating and a commercial fluorinated hydrophobic coating. SEM analysis suggests that the coatings arrange themselves following the morphology of the stones, while, the analysis of the degree of penetration, performed by means of micro-CT, shows that the coating remains on the surface.

Colorimetric analyses show that the treatments used do not alter the color of the stone materials, an essential characteristic for cultural heritage regulations.

Contact angle measurements demonstrate that the proposed coating significantly reduces surface wettability, ensuring impermeability: recorded contact angle values are comparable to those recorded with the commercial hydrophobic product and reach values > 140°. The coating ensures high resistance to capillary rise, as demonstrated by capillary absorption tests.

Vapor permeability, acting as an effective barrier against water, is significantly reduced but maintains values corresponding to medium water vapor transmittance. 

The coating is resistant to artificial aging; despite the stress, the stones still exhibit very high contact angle values, and their esthetic appearance is not altered by the photo-oxidative cycle.

All the analyses reported in this work have allowed us to evaluate the interaction of the proposed treatment with the surface of the chosen substrates and, on the basis of the results obtained, it can be confirmed that this protective treatment is promising for a possible application in the conservation of porous stone materials.

## Figures and Tables

**Table 1:** Description of the analyses/tests performed, number and size of the samples investigated, number of measurements per sample and the standard rules followed.

Analyses/Tests	Number of Samples	Dimensions (cm)	Measurements per Sample	Standard Rules
Petrographic analysis	1 untreated	3 × 1.5 × 0.003	1	
SEM-EDS	1 untreated 1 **HyB** 1 **HyC**	1.5 × 1.5 × 0.5	1	
Raman spectroscopy	1 untreated	1.5 × 1.5 × 1	5	
FTIR spectroscopy	1 untreated	powder	5	
X-ray micro-tomography	** 1 **HyA** + BaCl_2_	1.5 × 1.5 × 1.5	1	
* Colorimetric test	** 5 HyB ** 5 **HyC**	5 × 5 × 1	6	UNI EN 15886:2010 [49]
* Static contact angle	** 5 HyB ** 5 **HyC**	5 × 5 × 1	6	UNI EN 15802:2010 [50]
* Capillary water absorption	5 untreated 5 **HyB** 5 **HyC**	5 × 5 × 1	1	UNI EN 15801:2010 [51]
* Water vapor permeability	5 untreated 5 **HyB** 5 **HyC**	5 × 5 × 1	1	UNI EN 15803:2010 [52]

* The tests were performed sequentially on the same set of samples. ** Measurements were carried out on the same sample before and after treatments.

**Table 2:** Artificial aging: number of samples analyzed, number of measurements per sample, and tests performed.

* Samples	Number of Samples	Measurements per Sample	Tests
**HyB**	3	6	Colorimetric measurements
**HyC**	3	6	
**HyB**	3	6	Static contact angle
**HyC**	3	6	

* The tests were performed sequentially on the same set of samples.

**Table 3:** Summary of chromatic change (ΔE*), contact angle, water absorption coefficient (*AC*), water vapor transmission rate (*WVTR*) and reduction percentage vapor permeability (*RVP*%). The standard deviations are reported. The tests were performed sequentially on the same set of samples. The ΔE* and contact angle measurements were carried out on the same samples before and after treatments.

Samples	ΔE* (Averaged from 6 Points per Sample Before and After Treatment)	Contact Angle (°) (Averaged from 6 Drops per Sample Before and After Treatment)	*AC* (kg/(m^2^s^1/2^)) (Averaged from 5 Samples)	*WVTR* (g/(m^2^d)) (Averaged from 5 Samples)	*RVP* (%) (Averaged from 5 Samples)
Untreated	-	Not measurable	0.029 ± 0.001	130 ± 2	-
**HyB**	4.4 ± 1	140 ± 2	6.3 × 10^−4^ ± 2 × 10^−5^	76 ± 3	40 ± 2
**HyC**	3.7 ± 1	141 ± 2	1.0 × 10^−3^ ± 5 × 10^−4^	68 ± 2	48 ± 2

**Table 4:** The ΔE* and contact angle measurements recorded on **HyB**- and **HyC**-coated samples after aging.

Aged Samples		
Samples	ΔE* Before and After Aging (Averaged from 6 Points per Sample)	Contact Angle (°) (Averaged from 6 Drops per Sample)
**HyB**	2.1 ± 0.5	129 ± 2
**HyC**	1.5 ± 0.5	126 ± 2

## Data Availability

The original contributions presented in this study are included in the article. Further inquiries can be directed to the corresponding authors.

## References

[1] De la Torre M. (2013). Values and heritage conservation. Herit. Soc..

[2] Artesani A., Di Turo F., Zucchelli M., Traviglia A. (2020). Recent advances in protective coatings for cultural heritage–an overview. Coatings.

[3] Lettieri M., Masieri M., Aquaro M., Dilorenzo D., Frigione M. (2021). Eco-Friendly Protective Coating to Extend the Life of Art-Works and Structures Made in Porous Stone Materials. Coatings.

[4] Hosseini M., Karapanagiotis I. (2018). Advanced Materials for the Conservation of Stone.

[5] Raneri S., Barone G., Mazzoleni P., Alfieri I., Bergamonti L., De Kock T., Cnudde V., Lottici P.P., Lorenzi A., Predieri G. (2018). Efficiency assessment of hybrid coatings for natural building stones: Advanced and multi-scale laboratory investigation. Constr. Build. Mater..

[6] Ruffolo S.A., La Russa M.F. (2019). Nanostructured coatings for stone protection: An overview. Front. Mater..

[7] Yan Y., Wang Y. (2024). A Review of Atmospheric Deterioration and Sustainable Conservation of Calcareous Stone in Historical Buildings and Monuments. Sustainability.

[8] Benavente D., Cultrone G., Gómez-Heras M. (2008). The combined influence of mineralogical, hygric and thermal properties on the durability of porous building stones. Eur. J. Mineral..

[9] Salvini S., Coletti C., Maritan L., Massironi M., Pieropan A., Spiess R., Mazzoli C. (2023). Petrographic characterization and durability of carbonate stones used in UNESCO World Heritage sites in northeastern Italy. Environ. Earth Sci..

[10] Calvo J.P., Regueiro M. (2010). Carbonate rocks in the Mediterranean region—From classical to innovative uses of building stone. Geol. Soc. Lond. Spéc. Publ..

[11] Cioban L.A., Dochia M., Muresan C., Chambre D.R. (2022). Weathering and deterioration of carbonate stones from historical monuments: A review. Sci. Tech. Bull. Ser. Chem. Food Sci. Eng..

[12] Benavente D., de Jongh M., Cañaveras J.C. (2021). Weathering Processes and Mechanisms Caused by Capillary Waters and Pigeon Droppings on Porous Limestones. Minerals.

[13] Isola D., Bartoli F., Meloni P., Caneva G., Zucconi L. (2022). Black Fungi and Stone Heritage Conservation: Ecological and Metabolic Assays for Evaluating Colonization Potential and Responses to Traditional Biocides. Appl. Sci..

[14] Cardell C., Benavente D., Rodríguez-Gordillo J. (2008). Weathering of limestone building material by mixed sulfate solutions. Characterization of stone microstructure, reaction products and decay forms. Mater. Charact..

[15] Eyssautier-Chuine S., Marin B., Thomachot-Schneider C., Fronteau G., Schneider A., Gibeaux S., Vazquez P. (2016). Simulation of acid rain weathering effect on natural and artificial carbonate stones. Environ. Earth Sci..

[16] Doehne E., Price C.A. (2010). Stone Conservation—An Overview of Current Research.

[17] Fassina V. (1988). Environmental pollution in relation to stone decay. Durab. Build. Mater..

[18] Ruffolo S.A., La Russa M.F., Rovella N., Ricca M. (2023). The Impact of Air Pollution on Stone Materials. Environments.

[19] Maravelaki-Kalaitzaki P., Kallithrakas-Kontos N., Agioutantis Z., Maurigiannakis S., Korakaki D. (2008). A comparative study of porous limestones treated with silicon-based strengthening agents. Prog. Org. Coat..

[20] Lettieri M., Masieri M. (2016). Performances and coating morphology of a siloxane-based hydrophobic product applied in different concentrations on a highly porous stone. Coatings.

[21] Corcione C.E., De Simone N., Santarelli M.L., Frigione M. (2017). Protective properties and durability characteristics of experimental and commercial organic coatings for the preservation of porous stone. Prog. Org. Coat..

[22] Calò G., Di Pierro M., Federico A., Mongelli G. (1985). Caratteri geologici petrografici mineralogici e meccanici dei carpari della provincia di Lecce. Quarry and Construction.

[23] La Verde G., Raulo A., D’Avino V., Paternoster G., Roca V., La Commara M., Pugliese M. (2021). Pietra leccese and other natural stones in puglia region: A new category of building materials for radiation protection?. Int. J. Environ. Res. Public Health.

[24] Congedo P.M., Baglivo C., D’Agostino D., Quarta G., Di Gloria P. (2021). Rising Damp in Building Stones: Numerical and Experimental Comparison in Lecce Stone and Carparo under Controlled Microclimatic Conditions. Constr. Build. Mater..

[25] Folk R.L. (1959). Practical petrographic classification of limestones. Bull. Am. Assoc. Petrol. Geol..

[26] Calia A., Lettieri M., Mecchi A., Quarta G. (2016). The role of the petrophysical characteristics on the durability and conservation of some porous calcarenites from Southern Italy. Geol. Soc. Spec. Publ..

[27] Fico D., De Benedetto G.E. (2019). Characterisation of surface finishes on ancient historical buildings in Salento (Southern Italy): An integrated analytical approach to establish the secrets of artisans. J. Cult. Herit..

[28] Andriani G.F., Walsh N. (2010). Petrophysical and mechanical properties of soft and porous building rocks used in Apulian monuments (south Italy). Geol. Soc. Lond. Spec. Publ..

[29] Fico D. (2015). Càrparu e Leccisu: Tecniche di Finitura e Deterioramento Degli Edifici Storici del Salento. Ph.D. Thesis.

[30] Licchelli M., Malagodi M., Weththimuni M.L., Zanchi C. (2013). Water-repellent properties of fluoroelastomers on a very porous stone: Effect of the application procedure. Prog. Org. Coat..

[31] Bergamonti L., Potenza M., Scigliuzzo F., Meli S., Casoli A., Lottici P.P., Graiff C. (2024). Hydrophobic and Photocatalytic Treatment for the Conservation of Painted Lecce stone in Outdoor Conditions: A New Cleaning Approach. Appl. Sci..

[32] Zhang H., Liu Q., Liu T., Zhang B. (2013). The preservation damage of hydrophobic polymer coating materials in conservation of stone relics. Prog. Org. Coat..

[33] Sabatini V., Pargoletti E., Comite V., Ortenzi M.A., Fermo P., Gulotta D., Cappelletti G. (2019). Towards Novel Fluorinated Methacrylic Coatings for Cultural Heritage: A Combined Polymers and Surfaces Chemistry Study. Polymers.

[34] Wang P., Wei W., Li Z., Duan W., Han H., Xie Q. (2020). A superhydrophobic fluorinated PDMS composite as a wearable strain sensor with excellent mechanical robustness and liquid impalement resistance. J. Mater. Chem. A.

[35] Petcu C., Alexandrescu E., Bălan A., Tănase M.A., Cinteză L.O. (2020). Synthesis and characterisation of organo-modified silica nanostructured films for the water-repellent treatment of historic stone buildings. Coatings.

[36] Henry B.J., Carlin J.P., Hammerschmidt J.A., Buck R.C., Buxton L.W., Fiedler H., Seed J., Hernandez O. (2018). A critical review of the application of polymer of low concern and regulatory criteria to fluoropolymers. Integr. Environ. Assess. Manag..

[37] Kapridaki C., Maravelaki-Kalaitzaki P. (2013). TiO_2_–SiO_2_–PDMS nano-composite hydrophobic coating with self-cleaning properties for marble protection. Prog. Org. Coat..

[38] Manoudis P.N., Karapanagiotis I. (2014). Modification of the wettability of polymer surfaces using nanoparticles. Prog. Org. Coat..

[39] Manoudis P.N., Tsakalof A., Karapanagiotis I., Zuburtikudis I., Panayiotou C. (2009). Fabrication of super-hydrophobic surfaces for enhanced stone protection. Surf. Coat. Technol..

[40] Karapanagiotis I., Pavlou A., Manoudis P.N., Aifantis K.E. (2014). Water repellent ORMOSIL films for the protection of stone and other materials. Mater. Lett..

[41] Mosquera M.J., de los Santos D.M., Rivas T. (2010). Surfactant-synthesized ormosils with application to stone restoration. Langmuir.

[42] Li D., Xu F., Liu Z., Zhu J., Zhang Q., Shao L. (2013). The effect of adding PDMS-OH and silica nanoparticles on sol–gel properties and effectiveness in stone protection. Appl. Surf. Sci..

[43] Kapridaki C., Pinho L., Mosquera M.J., Maravelaki-Kalaitzaki P. (2014). Producing photoactive, transparent and hydrophobic SiO_2_-crystalline TiO_2_ nanocomposites at ambient conditions with application as self-cleaning coatings. Appl. Catal. B.

[44] Zarzuela R., Carbú M., Gil A., Cantoral J., Mosquera M.J. (2021). Incorporation of functionalized Ag-TiO_2_NPs to ormosil-based coatings as multifunctional biocide, superhydrophobic and photocatalytic surface treatments for porous ceramic materials. Surf. Interfaces.

[45] Nguyen T.V., Nguyen T.A., Dao P.H., Mac V.P., Nguyen A.H., Do M.T. (2016). Effect of rutile titania dioxide nanoparticles on the mechanical property, thermal stability, weathering resistance and antibacterial property of styrene acrylic polyurethane coating. Adv. Nat. Sci. Nanosci. Nanotechnol..

[46] Bergamonti L., Bondioli F., Alfieri I., Alinovi S., Lorenzi A., Predieri G., Lottici P.P. (2018). Weathering resistance of PMMA/SiO_2_/ZrO_2_ hybrid coatings for sandstone conservation. Polym. Degrad. Stab..

[47] Lacan P., Guizard C., Cot L. (1995). Chemical and rheological investigations of the sol-gel transition in organically-modified siloxanes. J. Sol-Gel Sci. Technol..

[48] (2001). Conservation of Cultural Property—Test Methods—Natural and Artificial Stone Materials—Hydrophobic Products.

[49] (2010). Conservation of Cultural Property—Test Methods—Color Measurement of Surfaces.

[50] (2010). Conservation of Cultural Property—Test Methods—Determination of Static Contact Angle.

[51] (2010). Conservation of Cultural Property—Test Methods—Determination of Water Absorption by Capillarity.

[52] (2010). Conservation of Cultural Property—Test Methods—Determination of Water Vapour Permeability.

[53] Washburn E.W. (1921). The dynamics of capillary flow. Phys. Rev..

[54] Wang H., Xu P., Zhong W., Shen L., Du Q. (2005). Transparent poly (methyl methacrylate)/silica/zirconia nanocomposites with excellent thermal stabilities. Polym. Degrad. Stab..

[55] Wang X., Wu L., Li J. (2010). Influence of nanozirconia on the thermal stability of poly (methyl methacrylate) prepared by in situ bulk polymerization. J. Appl. Polym. Sci..

[56] Bergamonti L., Verza E., Magnani R., Michelini E., Ferretti D., Lottici P.P., Graiff C. (2025). Protection of gypsum artifacts by Mg(OH)_2_ based super-hydrophobic nanocomposite. Constr. Build. Mater..

[57] Téllez L., Rubio J., Rubio F., Morales E., Oteo J.L. (2004). FT-IR study of the hydrolysis and polymerization of tetraethyl orthosilicate and polydimethyl siloxane in the presence of tetrabutyl orthotitanate. Spectrosc. Lett..

[58] Xu F., Liu H., Li D. (2016). Synthesis of PDMS-SiO_2_ hybrids using different templates. J. Porous Mater..

[59] Bellido-Aguilar D.A., Zheng S., Zhan X., Huang Y., Zhao X., Zeng X., Pallathadka P.K., Zhang Q., Chen Z. (2019). Effect of a fluoroalkyl-functional curing agent on the wettability, thermal and mechanical properties of hydrophobic biobased epoxy coatings. Surf. Coat. Technol..

[60] Schramm C. (2020). High temperature ATR-FTIR characterization of the interaction of polycarboxylic acids and organotrialkoxysilanes with cellulosic material. Spectrochim. Acta A Mol. Biomol. Spectrosc..

[61] Wang Y.T., Chang T.C., Hong Y.S., Chen H.B. (2003). Effect of the interfacial structure on the thermal stability of poly (methyl methacrylate)–silica hybrids. Thermochim. Acta.

[62] Criado M., Sobrados I., Sanz J. (2014). Polymerization of hybrid organic–inorganic materials from several silicon compounds followed by TGA/DTA, FTIR and NMR techniques. Prog. Org. Coat..

[63] Camino G., Lomakin S.M., Lazzari M. (2001). Polydimethylsiloxane thermal degradation Part 1. Kinetic aspects. Polymer.

[64] Camino G., Lomakin S.M., Lageard M. (2002). Thermal polydimethylsiloxane degradation. Part 2. The degradation mechanisms. Polymer.

[65] Wen J., Mark J.E. (1995). Sol–gel preparation of composites of poly (dimethylsiloxane) with SiO_2_ and SiO_2_/TiO_2_, and their mechanical properties. Polym. J..

[66] Karlina L., Azmiyawati C., Darmawan A. (2019). Synthesis and characterization of hydrophobic silica prepared by different acid catalysts. IOP Conf. Ser. Mater. Sci. Eng..

[67] Gunasekaran S., Anbalagan G., Pandi S. (2006). Raman and infrared spectra of carbonates of calcite structure. J. Raman Spectrosc..

[68] Gunasekaran S., Anbalagan G. (2007). Spectroscopic characterization of natural calcite minerals. Spectrochim. Acta Mol. Biomol. Spectrosc..

[69] Bugani S., Camaiti M., Morselli L., Van de Casteele E., Janssens K. (2008). Investigating morphological changes in treated vs. untreated stone building materials by x-ray micro-CT. Anal. Bioanal. Chem..

[70] Callow B., Falcon-Suarez I., Marin-Moreno H., Bull J.M., Ahmed S. (2020). Optimal X-ray micro-CT image based methods for porosity and permeability quantification in heterogeneous sandstones. Geophys. J. Int..

[71] Toniolo L., Poli T., Castelvetro V., Manariti A., Chiantore O., Lazzari M. (2002). Tailoring new fluorinated acrylic copolymers as protective coatings for marble. J. Cult. Herit..

[72] Corcione C.E., Striani R., Frigione M. (2014). Novel hydrophobic free-solvent UV-cured hybrid organic–inorganic methacrylic-based coatings for porous stones. Prog. Org. Coat..

[73] García O., Malaga K. (2012). Definition of the procedure to determine the suitability and durability of an anti-graffiti product for application on cultural heritage porous materials. J. Cult. Herit..

